# A Case Study of Tai Chi Exercise Prescription and Active Health Community Development at Peking University

**DOI:** 10.1093/geroni/igaf122.1827

**Published:** 2025-12-31

**Authors:** Dongmin Wang, Ning Kang, Gong Chen

**Affiliations:** Peking University, Beijing, Beijing, China (People’s Republic); Peking University, Beijing, Beijing, China (People’s Republic); Peking University, Beijing, Beijing, China (People’s Republic)

## Abstract

This study explores the role of scientific Tai Chi exercise in promoting active health within university communities, advancing the implementation of the “Integration of Sports and Public Health” model to support the Healthy China strategy. Tai Chi has demonstrated significant benefits in cardiovascular health, musculoskeletal function, metabolic processes, and delaying aging.

